# Combining gene expression profiling and machine learning to diagnose B-cell non-Hodgkin lymphoma

**DOI:** 10.1038/s41408-020-0322-5

**Published:** 2020-05-22

**Authors:** Victor Bobée, Fanny Drieux, Vinciane Marchand, Vincent Sater, Liana Veresezan, Jean-Michel Picquenot, Pierre-Julien Viailly, Marie-Delphine Lanic, Mathieu Viennot, Elodie Bohers, Lucie Oberic, Christiane Copie-Bergman, Thierry Jo Molina, Philippe Gaulard, Corinne Haioun, Gilles Salles, Hervé Tilly, Fabrice Jardin, Philippe Ruminy

**Affiliations:** 1 INSERM U1245, Centre Henri Becquerel, UNIROUEN, University of Normandie, Rouen, France; 2 Department of Biological Hematology, Rouen University Hospital, Rouen, France; 3 Department of Pathology, Centre Henri Becquerel, Rouen, France; 4 LITIS EA 4108, UNIROUEN, University of Normandie, Rouen, France; 5 Department of Hematology, IUCT - Oncopole, Toulouse, France; 6 Department of Pathology, Henri Mondor Hospital, APHP, Paris Est-Créteil (UPEC) University Faculty, UMR-S 955, INSERM, Créteil, France; 7 Department of Pathology, Necker-Enfants Malades Hospital, AP-HP, Centre-Université de Paris, Paris, France; 8 Department of Hematology, Henri Mondor University Hospital, APHP, Creteil, France; 9 Hospices Civils de Lyon, Department of Hematology, Université de Lyon, INSERM 1052, Lyon, France; 10 Department of Clinical Hematology, Centre Henri Becquerel, Rouen, France

**Keywords:** Cancer genomics, B-cell lymphoma

## Abstract

Non-Hodgkin B-cell lymphomas (B-NHLs) are a highly heterogeneous group of mature B-cell malignancies. Their classification thus requires skillful evaluation by expert hematopathologists, but the risk of error remains higher in these tumors than in many other areas of pathology. To facilitate diagnosis, we have thus developed a gene expression assay able to discriminate the seven most frequent B-cell NHL categories. This assay relies on the combination of ligation-dependent RT-PCR and next-generation sequencing, and addresses the expression of more than 130 genetic markers. It was designed to retrieve the main gene expression signatures of B-NHL cells and their microenvironment. The classification is handled by a random forest algorithm which we trained and validated on a large cohort of more than 400 annotated cases of different histology. Its clinical relevance was verified through its capacity to prevent important misclassification in low grade lymphomas and to retrieve clinically important characteristics in high grade lymphomas including the cell-of-origin signatures and the *MYC* and *BCL2* expression levels. This accurate pan-B-NHL predictor, which allows a systematic evaluation of numerous diagnostic and prognostic markers, could thus be proposed as a complement to conventional histology to guide the management of patients and facilitate their stratification into clinical trials.

## Introduction

Non-Hodgkin B-cell lymphomas (B-NHLs) are a group of mature B-cell malignancies that tend to mimic the various normal stages of B cells differentiation. These tumors show a high degree of heterogeneity and their classification requires skillful histological examination, usually completed by ancillary methods like immunohistochemical studies (IHC), immunoglobulin clonality assessment, flow cytometry, conventional cytogenetics, fluorescence in situ hybridization or next-generation DNA sequencing^1–4^. However, the risk of error in diagnosis remains higher in these tumors than in many other areas of pathology, supporting the need for expert secondary review.

Recently, low throughput quantitative RNA assays have proven their routine applicability in high grade B-NHLs classification^5–7^. These approaches circumvent the complexity of pan-genomic gene expression profiling, which is hardly applicable in a routine diagnostic setting, by focusing on limited sets of genes associated to well defined gene expression signatures. However, these assays only address a small part of the complexity of B-NHLs, limiting their application in clinical practice.

Here, to evaluate further the potential of such methods for lymphoma classification, we have addressed the capacity of a middle throughput gene expression signature to differentiate the seven major histological subtypes of B-NHLs. To facilitate the interpretation of the data we purposefully included many markers identified in the WHO classification of lymphoid tumors for their capacity to differentiate these pathologies^8^. We also overcome the difficulty of multiclass classification by implementing an artificial intelligence algorithm that we trained and validated using more than 400 cases annotated by experts hemato-pathologists. Together, our data illustrate how the combination of middle throughput gene expression profiling and machine learning could assist pathologists for the diagnosis of these complex tumors.

## Materials and methods

### Patients

Five hundred ten B-NHL biopsies were analyzed in this study, including 325 diffuse large B-cell lymphomas (DLBCL), 43 primary mediastinal B-cell lymphomas (PMBL), 55 follicular lymphomas (FL), 31 mantle cell lymphomas (MCL), 17 small lymphocytic lymphoma (SLL), 20 nodal or splenic marginal zone lymphomas (MZL), 11 extranodal marginal zone lymphomas of mucosa-associated lymphoid tissue (MALT) and 8 lymphoplasmacytic lymphomas (LPL). Three hundred sixty-six patients were diagnosed at a single institution (Centre Henri Becquerel, Rouen, France). Additional patients were recruited from the SENIOR (*n* = 96) (clinicaltrial.gov=NCT02128061) and RT3 (*n* = 48) (clinicaltrial.gov=NCT03104478) clinical trials. Sample list is available in Supplemental Table 1. All diagnoses were established according to the 2016 World Health Organization criteria by a panel of expert pathologists from the LYSA^8^. For all patients, from the LYSA clinical trials and from the Centre Henri Becquerel, written consents were obtained before analysis of their biopsy samples.

### RNA extraction

For CHB biopsies, RNA was extracted from FFPE samples using the Maxwell 16 system (Promega, Manheim, Germany) or, when available, from frozen tissues using the RNA NOW kit (Biogentex, Seabrook, TX). For the samples from the RT3 and SENIOR trials, RNAs were extracted from FFPE biopsies using the Siemens TPS and Versant reagents kit (Siemens Health Care Diagnostics, Erlangen, Germany).

### Gene selection

A panel of 137 gene expression markers was designed for this study, to address the expression of B-cell differentiation markers, therapeutic targets, and prognostic markers. We also included T cell and macrophage markers, along with genes involved in the anti-tumor immune response to analyze the contribution of the microenvironment. For each marker, a pair of RT-MLPA probes was designed across one exon-exon junction to avoid unspecific amplification of genomic DNA. Two pairs were designed for *AICDA*, *BCL6*, *MYC*, and *BCL2* to increase the accuracy of the assay. Additional probes were designed to evaluate the expression of various *IGH* transcripts, to detect some recurrent somatic point mutations, and to evaluate some virus infection status (Table 1).

**Table 1:**
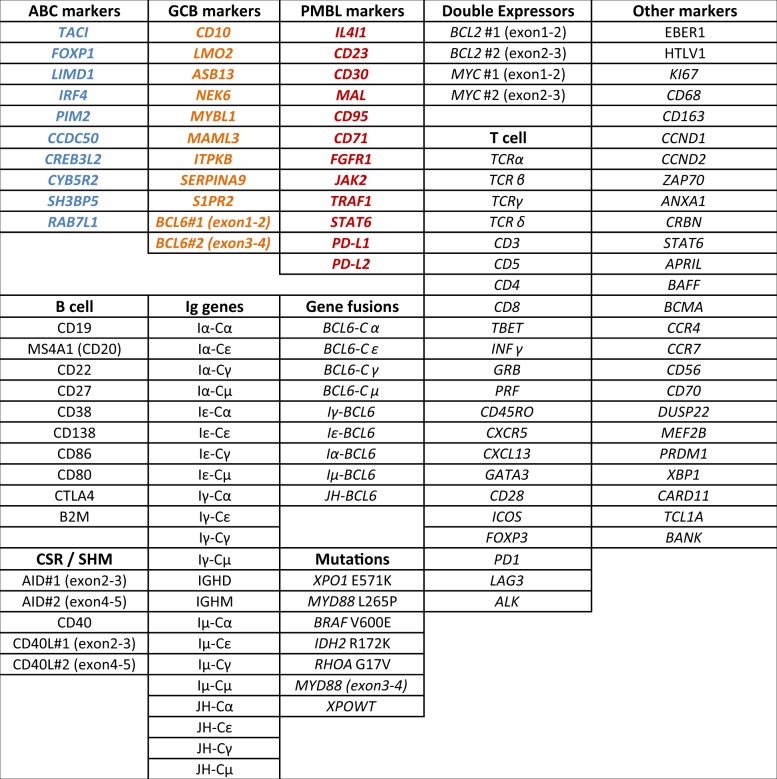
Markers included in the RT-MIS assay.

All 137 markers included in the assay are listed and bundled by groups. The panel includes B cell genes, Immunoglobulin genes, T cell genes, detection of recurrent somatic mutations, double expressors genes, ABC discriminating genes are labeled in blue, GCB genes in orange, PMBL genes in red and various other genes.

### Assay design and data processing

The RT-MLPSeq assay combines RT-MLPA and next-generation sequencing (NGS), as previously described^9^. Briefly, total RNAs, extracted from fresh or FFPE biopsies are quantified using a Qubit fluorometer (Thermo Fisher Scientific, Waltham, Massachusetts). Samples, with concentrations below 20 ng/µl, are excluded. Next, 50–200 ng RNA are converted into cDNA by reverse transcription using a M-MLV Reverse transcriptase and random hexamers to avoid 3’ end bias (Invitrogen, Carlsbad, CA). cDNA are incubated 1 h at 60 °C with a mix of ligation dependent PCR oligonucleotides probes, including universal adaptor sequences and random sequences of 7 nucleotides as unique molecular identifiers (UMI) in 1× SALSA MLPA buffer (MRC Holland, Amsterdam, the Netherlands), ligated using the thermostable SALSA DNA ligase (MRC Holland, Amsterdam, the Netherlands), and amplified by PCR using barcoded primers containing P5 and P7 adaptor sequences with the Q5 hotstart high fidelity master mix (NEB, Ipswich, MA). Amplification products are next purified using AMPure XP beads (Beckman Coulter, Brea, CA) and analyzed using a MiSeq sequencer (Illumina, San Diego, CA). Sequencing reads are de-multiplexed using the index sequences introduced during PCR amplification, aligned with the sequences of the probes and counted. All results are normalized according to the UMI sequences to avoid PCR amplification bias. Results are considered interpretable when at least 5000 different UMI (corresponding to the sum of all the UMI, for all the markers) are detected, allowing the evaluation of an average range of 1–40 for each marker.

### Statistical analysis

Correlations between immunohistochemical stainings and gene expression levels were evaluated using the Wilcoxon rank-sum test (two-sided). Differences in patient characteristics were evaluated using the *χ*^2^ or Fisher’s exact tests (two-sided) according to class size. Principal components analyses (PCAs) were built using the PCA function of FactomineR package in R software (http://www.r-project.org/). Genes that were significantly up- or downregulated between different conditions were analyzed using Welch’s unequal variances *t*-test procedure and visualized in volcano plots, plotting the significance versus log2-fold change on the *y* and *x* axes, respectively. Fold changes were computed as the base 2 logarithm of the mean change in the expression level of each gene between the two conditions. Genes with an absolute log2-fold change >1 and a significant FDR (<0.05) were plotted. Graphical representations were created using R software.

### Training of the machine learning algorithm

The training set was constructed using annotated B-NHL samples with one of the 7 following B-NHL subtypes: ABC DLBCL, GCB DLBCL, PMBL, FL, MCL, SLL, and MZL (regrouping MZL, MALT, and LPL). The random forest algorithm was next trained using the scikit-learn library for the Python programming language (Python Software Foundation, https://www.python.org/) using standard parameters (Gini index attribute selection criteria; max_depth, and min_samples_split set to 20, and 4, respectively). The obtained prediction model, which relies on 5000 different decision trees outputting the most likely B-NHL subtype was next applied to the independent validation sample set.

### Survival analyses

The survival of the 104 patients with DLBCL who were treated with a combination of rituximab and chemotherapy between 2000 and 2017 at the Centre Henri Becquerel was analyzed considering a risk of 5% as a significance threshold. Overall survival (OS) was computed from the day of treatment to death from any cause or right-censored at 5 years or the last follow-up. Progression-free survival (PFS) was computed from the day of treatment to disease progression, relapse, or death from any cause, or right-censored at 5 years or the last follow-up. Survival rates were estimated with the Kaplan–Meier method that provides 95% CIs, and significant differences between groups were assessed using the log-rank test. Different thresholds were tested to determine the ones that led to the most significant segmentation of patients and to evaluate the prognostic value of MYC, BCL2, and other markers. Those thresholds were subsequently combined to define the MYC+/BCL2+ double expression group. All analyses were performed using the Python survival package version 2.37.4.

## Results

### Technical validation

For validation, we first compared our method, which evaluate the expression of 137 genetic markers, with the Nanostring Lymph2Cx assay. As shown in Supplemental Fig. 1, linear correlations were observed for the 15 genes evaluated using the two methods applied to the 96 FFPE biopsy samples from the SENIOR clinical trial. Significant correlations with immunochemical staining was also obtained for the 48 DLBCL samples from the RT3 clinical trial (*CD10*, *BCL6, MUM1, MYC, BCL2*, and *Ki67*, reviewed by a panel of expert pathologists from the LYSA) (Supplemental Fig. 2), indicating excellent technical concordances.

### DLBCL COO assignment

**Fig. 1:**
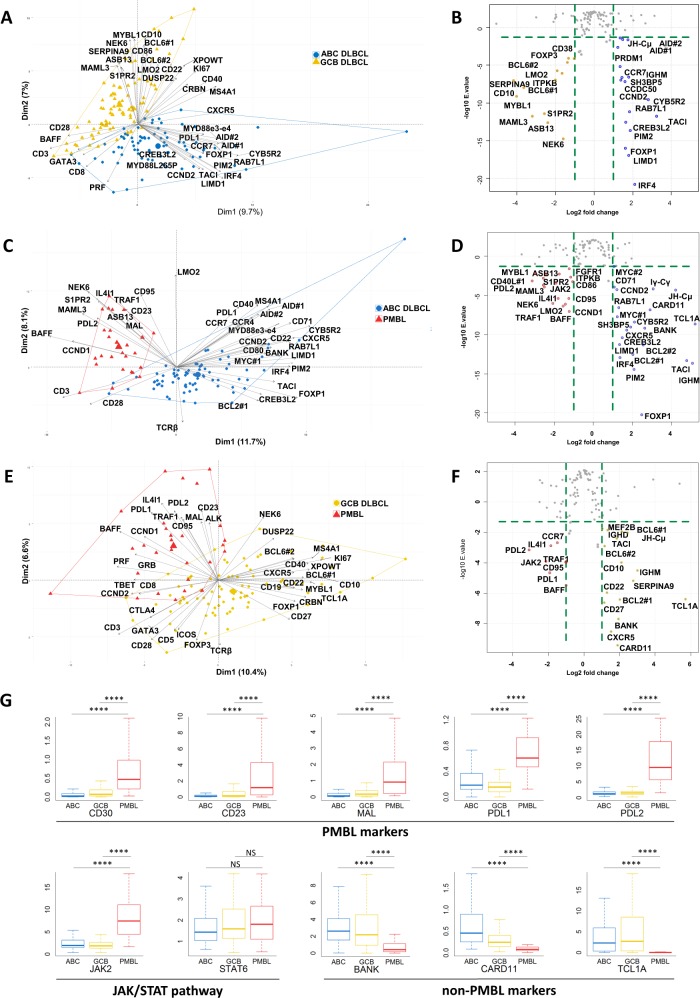
Transcriptomic expression analysis of diffuse large B-cell lymphoma. **a** Two-dimensional principal component analysis map computed on activated B-cell (ABC) DLBCL (blue) and germinal center B-cell (GCB) DLBCL (gold) cases for the 137 markers included in the panel. The expression of the 40 most discriminatory markers is plotted. **b** Volcano plots computed on ABC DLBCL (blue) and GCB DLBCL (gold) cases for the 137 markers included in the panel showing up- or downregulated genes between these 2 conditions (absolute log2-fold change >1 and a significant FDR (<0.05)). **c** Two-dimensional principal component analysis map computed on PMBL (red) and ABC DLBCL (blue) cases for the 137 markers included in the panel. The expression of the 40 most discriminatory markers is plotted. **d** Volcano plots computed on PMBL (red) and ABC DLBCL (blue) cases for the 137 markers included in the panel showing up- or downregulation between these 2 conditions (absolute log2-fold change >1 and a significant FDR (<0.05)). **e** Two-dimensional principal component analysis map computed on PMBL (red) and GCB DLBCL (gold) cases for the 137 markers included in the panel. The expression of the 40 most discriminatory markers is plotted. **f** Volcano plots computed on PMBL (red) and GCB DLBCL (gold) cases for the 137 markers included in the panel showing up- or downregulation between these two conditions (absolute log2-fold change >1 and a significant FDR (<0.05)). **g** Differential expression of a selection of markers of interest that is useful for distinguishing PMBL from ABC and GCB DLBCL. ****p < 10^−4^ and NS: not significant according to the Wilcoxon test.

We next addressed the ability of the panel of markers to discriminate the different subtypes of B-cell NHLs. We first tested capacity of the assay to recapitulate the COO classification of DLBCLs. As shown in Fig. 1, an unsupervised principal component analysis (PCA) and differential gene expression analysis (DGEA, volcano plot) of the 125 ABC and 127 GCB DLBCL cases from our cohort efficiently distinguished these two lymphoma subtypes (Fig. 1a), retrieving the expected gene expression signatures (Fig. 1b, Supplemental Table 2–3 and Supplemental Fig. 3). Interestingly, this analysis also identified a COO-independent T cell component (*CD28, BAFF, CD3, GATA3, CD8* and *PRF*) that probably reflects various levels of T cell infiltration in these tumors.

We next tested the capacity of the assay to discriminate PMBLs from other DLBCLs. The first components of the PMBL vs ABC and PMBL vs GCB PCA maps retrieved the three expected signatures (Fig. 1c, e). As shown in Fig. 1g, our results confirmed that the CD30 and CD23 markers, which are often evaluated using immunochemistry in the clinics for diagnostic purposes, were overexpressed at the RNA level in these samples. Our data were also consistent with the high expression of *PDL1*, *PDL2*, and *JAK2* and the downregulation of *BANK*, *CARD11*, and *TCL1A* reported in these tumors by Rosenwald et al.^10^.

### DLBCL/Small cell lymphoma classification

**Fig. 2:**
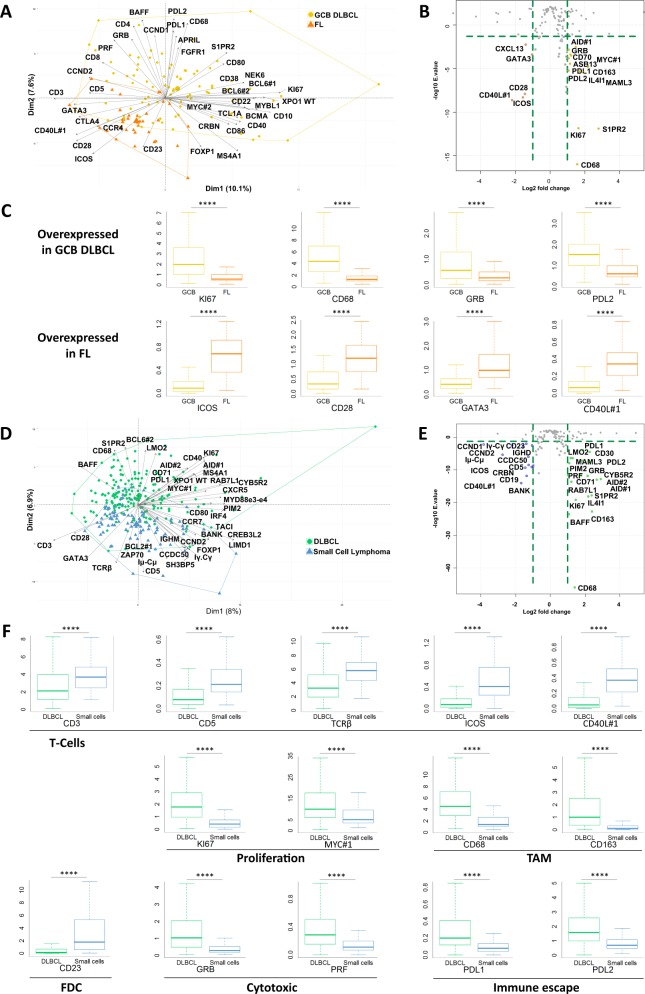
Differential transcriptomic analysis of diffuse large B-cell lymphoma and small cell lymphoma. **a** Two-dimensional principal component analysis map computed on GCB DLBCL (gold) and follicular lymphoma (orange) cases for the 137 markers included in the panel. The expression of the 40 most discriminatory markers is plotted. **b** Volcano plots computed on GCB DLBCL (gold) and follicular lymphoma (orange) cases for the 137 markers included in the panel showing up- or downregulation between these two conditions (absolute log2-fold change >1 and a significant FDR (<0.05)). **c** Differential expression of Ki67, the macrophage marker CD68, GRB, immune escape marker PD-L2, and Tfh markers in GCB DLBCL and FL samples. ****p < 10^−4^ by the Wilcoxon test. **d** Two-dimensional principal component analysis map computed on DLBCL (green) and small cell lymphoma (blue) cases for the 137 markers included in the panel. The expression of the 40 most discriminatory markers is plotted. **e** Volcano plots computed on DLBCL (green) and small cell lymphoma (blue) cases for the 137 markers included in the panel showing up- or downregulation between these two conditions (absolute log2-fold change >1 and a significant FDR (<0.05)). **f** Differential expression of a selection of markers involved in proliferation and the immune response between DLBCL and small cell lymphomas. ****p < 10^−4^ by the Wilcoxon test.

We next addressed the classification ability of the markers expressed by cells in the microenvironment. We first compared GCB DLBCLs and FLs, two lymphomas that develop from germinal center B-cells^11^. As shown in Fig. 2a, the first dimensions of the PCA map identified three major components. The first, which is associated with GCB DLBCLs, essentially regrouped GCB markers (*CD10, MYBL1, NEK6*, and *BCL6*), probably reflecting the higher percentage of malignant cells in these tumors. As shown in Fig. 2b, c, GCB DLBCLs were also characterized by the expression of the KI67 proliferation marker, the tumor-associated macrophage (TAM) marker CD68, and cytotoxic and immune escape markers (*GRB*, *PD-L1*, and *PD-L2*). As expected, the second component of this PCA, which is associated with FLs, regrouped many T cell markers (*CD3, CD5, CD28, CTLA4, GATA3*, and *CCR4*)^12^. FLs also significantly overexpressed the Tfh markers *ICOS*, *CD40L*, and *CXCL13*, as well as *CD23*, that can be expressed either by tumoral cells or by follicular dendritic cells.

As shown in Fig. 2d–f, the same PCA and DGEA methods applied to the whole cohort of cases revealed that the high expression of *KI67*, germinal center-associated genes (*LMO2*, *BCL6*, *MAML3*, *S1PR2*, and *CD40*), the CD68 and CD163 TAM markers, the *GRZB* and *PRF* cytotoxic markers, and the *PD-L1* and *PD-L2* immune checkpoints inhibitors were a common characteristic of aggressive lymphomas, regardless of the COO classification. This observation probably reflects the high turnover of lymphoma cells within these tumors, together with the presence of scavenger cells and the existence of an active anti-tumor immune response^13,14^. Conversely, low-grade lymphoma were characterized by the expression of T cell markers (*CD3, CD5*, the beta chain of the *TCR, ICOS*, and *CD40L)* and a follicular dendritic cell marker (*CD23*), probably reflecting the crosstalk between lymphoma cells and their environment for survival and proliferation^12^.

### Small B-cell lymphoma classification

**Fig. 3:**
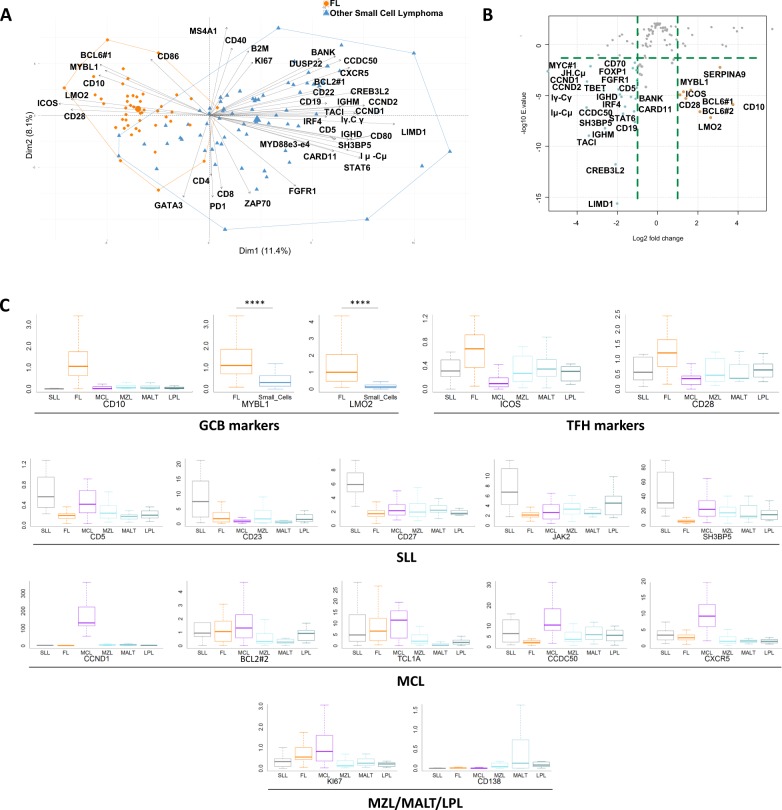
Transcriptomic expression analysis of small B-cell lymphoma. **a** Two-dimensional Principal Component Analysis map computed on small cell lymphoma cases, including follicular lymphoma (orange) and other small cell lymphoma (blue) cases, for the 137 markers included in the panel. The expression of the 40 most discriminatory markers is plotted. **b** Volcano plots computed on follicular lymphoma (orange) and other small cell lymphoma (blue) cases for the 137 markers included in the panel showing up- or downregulation between these two conditions (absolute log2-fold change >1 and a significant FDR (<0.05)). **c** Differential expression of a selection of GCB markers, Tfh markers and various markers of interest in the diagnosis of small cell lymphomas. ****p < 10^−4^ by the Wilcoxon test.

We next addressed the capacity of the assay to discriminate the different subtypes of small cell B-NHLs. As shown in Fig. 3a, the first dimensions of the PCA map restricted to low grade B-NHLs identified two major components. The first, which is associated with FLs, regrouped GCB (*BCL6*, *MYBL1*, *CD10*, and *LMO2*) and T cells markers (*CD28*, *ICOS*). The second regrouped many activated B-cell markers (*LIMD1, TACI, SH3BP5, CCDC50, IRF4*, and *FOXP1*), consistent with the late GC or memory B-cell origin of others small B-cell lymphomas^11^.

We next addressed the capacity of the assay to retrieve the main characteristics used in the clinics for the classification of these tumors (Fig. 3c). The *CD5pos*, *CD23pos*, *CD10neg* phenotype of SLLs was correctly identified^15^. Interestingly, these tumors also expressed *CD27*, consistent with their mature B-cell origin, *JAK2*, suggesting the activation of the JAK/STAT pathway^16^, and downregulated *SH3BP5*, indicating a possible negative regulatory effect on Bruton’s tyrosine kinase activity^17^. In MCLs, our assay retrieved the expected *CCND1*high, *CD5*high, and *BCL2*high phenotype, together with the expected downregulation of *CD10* and *CD23*. Interestingly, *TCL1A* and *CCDC50*, both of which are associated with survival in patients with this pathology^18,19^, and the B-cell chemokine receptor *CXCR5*, which is involved in dissemination^20^, were overexpressed in these tumors compared to other small B-cell NHLs. Finally, the MZL group showed the expected *CD5*neg, *CD10*neg, *CD23*neg phenotype, together with high expression of *CD138* and low expression of *Ki67*.

### *IGH* transcripts participate in the classification of B-NHLs

**Fig. 4:**
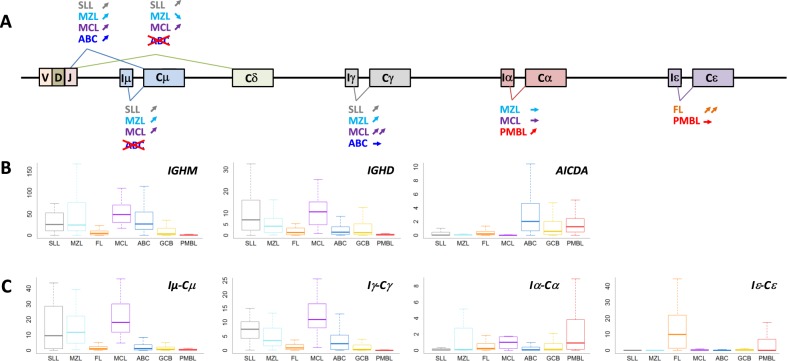
Analysis of immunoglobulin transcripts in B-NHLs. **a** Schematic of the regulation of immunoglobulin transcripts. Mature B-cells constitutively transcribe VDJ, Cµ, and Cδ encoding IgM and IgD. In the presence of specific sets of activation signals, B-cells initiate class switch recombination through the germline transcription of downstream Cγ, Cα or Cε genes. The expression of sterile transcripts required for class switching after *AICDA*-induced genetic instability is also displayed for different subtypes. **b** Differential expression of the immunoglobulin transcripts *IGHM* and *IGHD* and the expression of *AICDA* in the global cohort are plotted, showing an overexpression of IGHM in tumor cells from patients with SLL, MZL, MCL, and ABC DLBCL. **c** Differential expression of immunoglobulin sterile transcripts required for class switching are plotted, showing a high expression of *Iµ-Cµ* transcript in *IGHM*-positive tumors, for except ABC DLBCL, despite *AICDA* expression. The sterile transcript Iε-Cε is consistently and almost exclusively expressed in FL samples.

In addition to their cellular origin and the composition of their microenvironment, B-cell NHLs also differ in the configurations of their immunoglobulin genes. As shown in Fig. 4, MCL and SLL can be distinguished from other B-NHLs based on the expression of the *IGHD* gene. Two groups of tumors can also be defined according to the expression of the *IGHM* gene. The first corresponds to the *IGHM*-positive tumors with an activated or memory B-cell origin (most ABC DLBCLs, MCL, MZL, and SLL). The second corresponds to the tumors of GCB origin (particularly, GCB DLBCLs and FL), which often undergo isotype switching, and PMBLs, which usually lack immunoglobulin expression. Interestingly, our data also confirmed the existence of a class switch recombination (CSR) defect in ABC DLBCLs. As previously reported, our data confirmed that a majority of ABC DLBCLs paradoxically express the *IGHM* gene along with *AICDA*, a direct activator of immunoglobulin isotype switching^21,22^. We evaluated the expression of the immunoglobulin sterile transcripts required for CSR activation to clarify this issue and observed that the expression of *AICDA* and the *Iμ-Cμ* transcript, which controls the accessibility of the switch µ region to the CSR machinery, are specifically desynchronized in these tumors. This *Iμ-Cμ* transcript is expressed by a majority of IgM-positive NHLs (SLLs, MZLs, and MCLs), which do not express *AICDA*, but is downregulated in ABC DLBCLs, probably preventing isotype switching despite of *AICDA* expression. Surprisingly, we also observed that the Iγ-Cγ sterile transcript is expressed at a high level in SLL and MCL, two nongerminal center-derived lymphomas, and the Iε-Cε transcript is almost exclusively expressed in FLs constituting one of the most discriminatory markers for this pathology in our assay.

### Development of a random forest pan-B NHL classifier

We next trained a random forest (RF) classifier to discriminate the 7 principal subtypes of B-cell NHLs. DLBCLs with an ambiguous classification (inconclusive cell-of-origin classification by RT-MLPA and/or Nanostring Lymph2Cx), EBV-positive DLBCLs, and grade 3B FLs were excluded from the training. The 429 remaining cases were randomly assigned to a training cohort of 283 cases (two-thirds) and to a validation cohort of 146 cases (one-third). The training cohort comprised 190 DLBCLs (76 ABC, 86 GCB and 28 PMBL cases) that were previously classified by IHC and/or RT-MLPA, 35 FLs (grade 1–3 A), 21 MCLs, 12 SLLs, and 25 cases in the MZL category (13 MZLs, 8 MALT lymphomas and 4 LPLs). The validation series comprised the 90 DLBCLs from the SENIOR trial classified as GCB (41 cases) or ABC (49 cases) DLBCLs by the Nanostring Lymph2Cx assay, 15 PMBLs, 12 grade 1–3 A FLs, 10 MCLs, 5 SLLs, and 14 MZLs (7 MZL, 3 MALT, and 4 LPL). A schematic overview of these cohorts is presented in Supplemental Fig. 4.

**Fig. 5:**
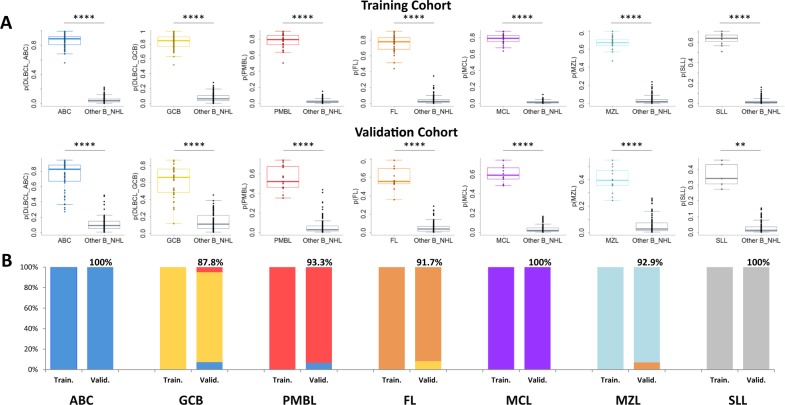
Results of the classification of the training and validation cohorts using the random forest algorithm. **a** Distribution of the random forest algorithm probabilities that a sample belongs to the expected class is plotted for each subtype in the training (*n* = 283) cohort, showing a significantly higher probability in the expected class. **b** Distribution of the random forest algorithm probabilities in the validation (*n* = 146) cohort. **c** Proportion of cases accurately classified by the random forest algorithm for each B-NHL subtype in the training and validation cohorts. ****p < 10^−4^ and **p < 0.01 by the Wilcoxon test.

The RF algorithm classified all 283 cases of the training series into the expected subtype. As shown in Fig. 5a, the distributions of the probabilities that the tumor belonged to one of the 7 subclasses indicated a very good capacity of the algorithm to discriminate these lymphomas. The RF predictor also classified 138/146 (94.5%) of the samples in the independent validation cohort into the expected subtype, showing a very good generalization capacity (Fig. 5b). For the ABC and GCB DLBCLs, the concordance with the Lymph2Cx assay in the validation cohort was 94.3%. Our method agreed with the Lympho2Cx assay for 49/49 (100%) ABC DLBCLs and 36/41 (87.8%) GCB DLBCLs. Two cases classified as GCB DLBCLs by the Lymph2Cx assay were classified as PMBL by the RF predictor. Further analyses of these two cases identified genomic mutations compatible with the PMBL diagnosis, which is not addressed by the Lymph2Cx assay (*B2M*, *TNFRSF14*, *SOX11*, and *CIITA* mutations for one case; *STAT6*, *B2M*, *CD58*, *CIITA*, and *CARD11* mutations for the other)^23^. The three other discordant cases were classified as ABC by the RF predictor, but no COO-specific mutations were detected in these samples. Notably, 14/15 PMBLs (93.3%) and 39/41 (95.1%) small cell lymphomas in the validation cohort were accurately classified, including all MCLs and SLLs. One MZL was classified as a FL, probably due to its preeminent GCB signature, and one FL was classified as a GCB DLBCL. For this patient, who presented with a stage IV lymphoma and a leukemic presentation, the examination of the gene expression values showed that the genes of the GCB signature and *BCL2* were highly expressed in the tumor, suggesting a high tumor/microenvironment ratio and further pointing to the need for a rigorous initial histological evaluation for classification. Interestingly, 5 of the 8 FL3B tumors, which we had excluded from the model building, were classified as DLBCLs by the RF predictor (3 GCB and 2 ABC cases), while 3 were classified as FLs. Otherwise, 5 of the 6 DLBCLs defined as unclassified by the Lymph2Cx assay were classified as ABC DLBCLs, including two samples harboring a *CD79B* mutation, which is usually associated with the ABC signature, and the last case was classified as GCB DLBCL, without COO-specific mutations detected (*ARID1A* and *CDKN2A*).

### DLBCL survival analyses

**Fig. 6:**
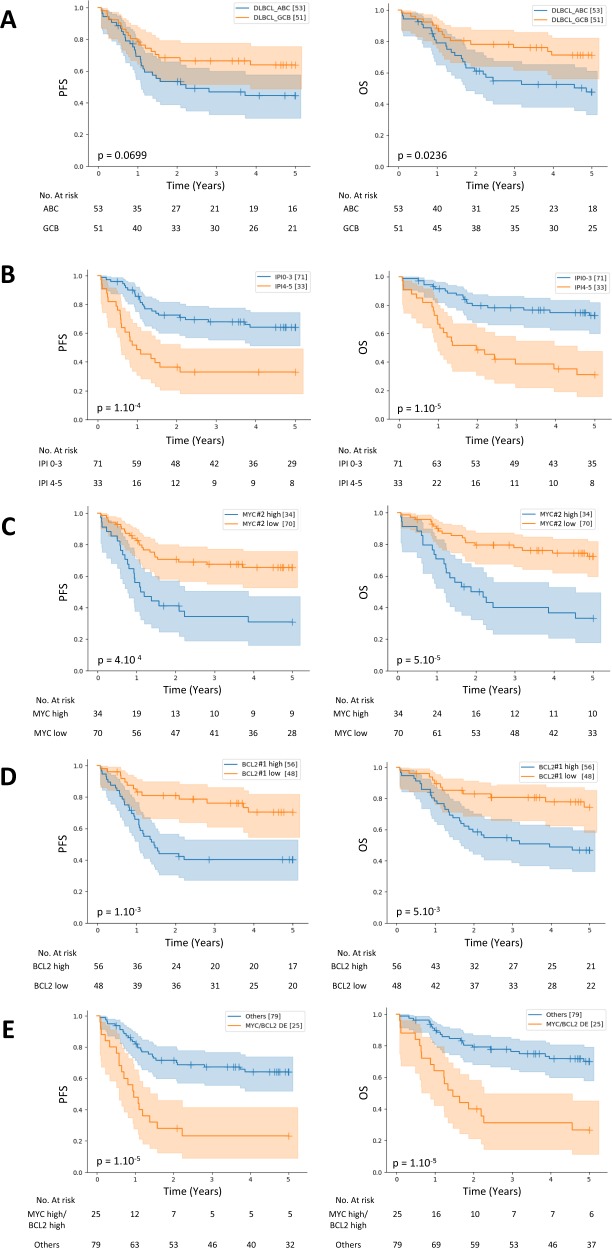
Progression-free survival (PFS) and overall survival (OS) in patients with DLBCL treated with rituximab plus chemotherapy from a local cohort stratified according to GCB/ABC cell-of-origin, IPI status, MYC or BCL2 expression and combined MYC/BCL2 expression. Survival curves for 104 patients from the local cohort stratified according to **a** GCB or ABC cell-of-origin determined by the random forest predictor, **b** IPI status, **c** MYC status determined by gene expression profiling, **d** BCL2 status determined by gene expression profiling, or **e** combined MYC/BCL2 double expression status.

We next focused on the 104 patients with DLBCL who were treated with a combination of rituximab and chemotherapy at the Centre Henri Becquerel to further evaluate the clinical value of the assay. In this cohort, the ABC/GCB COO was associated with OS (*p* = 0.0236), but only a trend was observed with PFS (*p* = 0.0699) (Fig. 6a and Supplemental Fig. 5). As shown in Fig. 6b–d, IPI status, *MYC* expression, and *BCL2* expression were associated with poorer PFS and OS. The combination of a high expression of *MYC* and *BCL2* identified a group of double-positive cases (24% of patients) with a particularly poor outcome (PFS, *p* < 10^–4^ and OS, *p* < 10^−4^) (Fig. 6e). This observation was confirmed with a multivariable model adjusted for the IPI score and cell-of-origin classification for both OS (HR, 2.08, 95% CI, 1.34–3.25, *p* < 5 × 10^−3^) and PFS (HR, 2.04, 95% CI, 1.35–3.12, *p* < 5 × 10^−3^), independent of the IPI (OS HR, 2.20, 95% CI, 1.41 to 3.41, *p* < 5 × 10^−3^; PFS HR, 1.92, 95% CI, 1.27 to 2.89, *p* < 5 × 10^−3^) (Table 2). Clinical and biological characteristics of these patients, presented in Supplemental Table 4, identified significant correlations between the MYC/BCL2 double-positive status and higher age (*p* = 5 × 10^−3^), elevated LDH levels (*p* = 0.04) and ABC subtype (*p* < 10^−4^), in accordance with previous studies^24,25^. As shown in Supplemental Fig. 6, the expression of other genes was also strongly correlated with PFS and OS in this cohort, including *CARD11* (PFS, *p* < 10^−3^ and OS, *p* < 10^−4^), *CREB3L2* (PFS, *p* < 10^−4^ and OS, *p* < 10^−4^), *STAT6* (PFS, *p* < 10^−3^ and OS, *p* < 10^−2^) and *CD30* (PFS, *p* < 10^−2^ and OS, *p* < 10^−3^).

**Table 2:** Multivariate analysis of MYC/BCL2 dual expression, ABC/GCB Cell-of-origin and IPI in local cohort of DLBCL.

	Overall survival			Progression-free survival		
Factor	HR	95% CI	*P*	HR	95% CI	*P*
MYC/BCL2 Double expressor (*n* = 25) vs other (*n* = 79)	2.08	1.34–3.25	<5 × 10^−^^3^	2.04	1.35–3.12	<5 × 10^−3^
ABC (*n* = 53) vs GCB (*n* = 51) subtype	1.49	0.95–2.36	0.08	1.32	0.87–2.00	0.19
IPI score 3–5 (*n* = 71) vs IPI score 0–2 (*n* = 33)	2.2	1.41–3.41	<5 × 10^−3^	1.92	1.27–2.89	<5 × 10^−3^

## Discussion

In this study, we developed a robust middle throughput gene expression assay to classify the seven most frequent subtypes of B-cell NHLs defined in the WHO classification. Previous studies have shown that these tumors can be defined according to the gene expression signatures of the tumoral cells and of their microenvironment, but a method able to differentiate such a variety of lymphoma in a single experiment is not yet available.

This assay relies on the evaluation of the RNA expression of more than 130 markers through a rapid four steps procedure which requires only very limited laboratory handling (reverse transcription, hybridization of the probes, ligation, and PCR amplification). The amplification products are next purified and loaded on a next-generation sequencer, alone or together with conventional DNA libraries, and the results are automatically analyzed using a dedicated bioinformatic pipeline which returns the results of the random forest classification algorithm. The whole procedure thus does not require any specific platform and could be implemented in many molecular diagnostic laboratories which have already adopted next-generation sequencing in their routine diagnostic workflow.

One limitation of this assay is the need for a rigorous histological evaluation of the biopsy to distinguish reactive lymph nodes and other pathologies and to verify the consistency of the classification. However, once the hypothesis of B-NHL has been drawn, the gene expression data which are generated provide many relevant information through the systematic evaluation of dozens of diagnostic and prognostic markers. As we deliberately incorporated most differentiation markers identified in the WHO classification for their capacity to differentiate these tumors, the test is able to recognize essential B-NHLs characteristics, such as the COO gene expression signatures, together with the different contributions of the microenvironment. The results also allow direct comparisons with other methods already in use in the clinics, like IHC or flow cytometry, greatly facilitating their interpretation.

In high grade B-NHLs the clinical value of this assay was validated by the demonstration of its capacity to capture essential characteristics associated with the prognosis, such as the MYC/BCL2 expression levels which can be complex to address due to the difficulties in the standardization of the IHC procedures. Indeed, the cut-offs values for MYC and BCL2 positivity evaluated by pathologists are still a matter of debate, but recent studies reported prevalence of double expressor DLBCL cases ranging from 21 to 31%^24,26–29^. In our cohort, 24% of patients were identified as double-positive according to their gene expression profiles and, importantly, were associated with a particularly poor outcome. This result thus suggests that the gene expression levels which are provided by our assay may represent a reliable surrogate to IHC as well as a good indicator of outcome, and help to identify those patients who may respond to targeted therapies^30^. Additional testing by FISH remains however mandatory to address which of these patients present chromosomal rearrangements of these genes and should be classified into the high-grade B-cell lymphoma category.

The limited panel of genes we use of course does not provide an exhaustive picture of the heterogeneity of these tumors. For example, new molecular subgroups of DLBCLs have recently been defined by extensive genomic and transcriptomic analyses which could, in the future, participate to the development of tailored personalized medicine. However, if these results undoubtedly provide important new insights into the biology of these tumors, their current applicability in the clinics remains a significant issue, warranting further investigation in prospective trials. Moreover, their implementation in a routine diagnosis workflow would also need to resolve important technical and financial issues, which are not sustainable by a large majority of diagnostic platforms.

In conclusion, we have developed a complete gene expression assay that combines ligation-dependent PCR, NGS, and machine learning to classify B-cell lymphoma subtypes. We propose that this assay, which does not require any specific platform and can be applied to FFPE biopsies, could be used as an independent additional diagnostic tool to conventional histology and facilitate the classification of B-cell lymphoma by expert hematopathologist. It might thus result in a significant simplification of the diagnostic procedures by reducing the number of immunostainings. Its coordinate implementation with next-generation DNA sequencing, which requires the same platform, might improve precision diagnosis in these heterogeneous tumors.

## Supplementary information

Supplementary information

Supplemental Table 1
