# Supplementary material for: Combining gene expression profiling and machine learning to diagnose B-cell non-Hodgkin lymphoma
Source: Blood Cancer J. 2020 May 22;10(5):59. doi: 10.1038/s41408-020-0322-5 (PMC7244768; doi:10.1038/s41408-020-0322-5)

**Combining Gene Expression Profiling and Artificial Intelligence to**

**Diagnose B-Cell non-Hodgkin Lymphoma**

*Bobée et al.*

**Supplemental Data**

Summary

[Supplemental Methods 2](#_Toc35447969)

[Robustness of the gene expression assay 2](#_Toc35447970)

[Supplemental Tables 3](#_Toc35447971)

[Supplemental Table 1 Sample list, IHC and gene expression data 3](#_Toc35447972)

[Supplemental Table 2 Top differentially expressed genes according to the two first components of PCA maps 3](#_Toc35447973)

[Supplemental Table 3 Significantly overexpressed genes and corresponding E-values for each Volcano plot 6](#_Toc35447974)

[Supplemental Table 4 Clinical and Biological characteristics of local DLBCL cases according to MYCBCL2+ status 10](#_Toc35447975)

[Supplemental Figures 11](#_Toc35447976)

[Supplemental Figure 1 Comparison of Nanostring nCounter and gene expression data 11](#_Toc35447977)

[Supplemental Figure 2 Comparison of IHC results and gene expression data 12](#_Toc35447978)

[Supplemental Figure 3 Transcriptomic expression of the markers from the ABC and GCB signatures in DLBCL 13](#_Toc35447979)

[Supplemental Figure 4 Schematic overview of the study design 14](#_Toc35447980)

[Supplemental Figure 5 Progression-free survival (PFS) and overall survival (OS) in patients with DLBCL treated with rituximab plus chemotherapy from a local cohort stratified according to GCB/ABC/PMBL cell-of-origin 16](#_Toc35447981)

[Supplemental Figure 6 Progression-free survival (PFS) and overall survival (OS) of patients with DLBCL treated with rituximab plus chemotherapy from a local cohort stratified according to CARD11, CREB3L2, STAT6 and CD30 expression 17](#_Toc35447982)

# Supplemental Methods

## Robustness of the gene expression assay

The assay was considered interpretable for 92.7% B-NHL samples (510/550). All samples that failed had less than 8 ng/µL RNA, as detected using a Qubit fluorometer (Thermo Fisher Scientific, Waltham, Massachusetts), and all results were interpretable when the concentration was greater than 8 ng/µL.

# Supplemental Tables

## Supplemental Table 1 Sample list, IHC and gene expression data

See in a different file (Supplemental Table 1.xlsx)

## Supplemental Table 2 Top differentially expressed genes according to the two first components of PCA maps

| **ABC DLBCL vs GCB DLBCL** | | | |
| --- | --- | --- | --- |
| **Principal Component 1** | | **Principal Component 2** | |
| *Positive* | *Negative* | *Positive* | *Negative* |
| 1. CYB5R2 | 1. CD3 | 1. CD10 | 1. PRF |
| 2. AID#1 | 2. CD28 | 2. MYBL1 | 2. LIMD1 |
| 3. LIMD1 | 3. BAFF | 3. NEK6 | 3. GRB |
| 4. RAB7L1 | 4. CD40L#1 | 4. BCL6#1 | 4. IRF4 |
| 5. IRF4 | 5. CD4 | 5. SERPINA9 | 5. TACI |
| 6. AID#2 | 6. TCRγ | 6. CD86 | 6. CCND2 |
| 7. MYD88e3-e4 | 7. GATA3 | 7. BCL6#2 | 7. LAG3 |
| 8. PIM2 | 8. FOXP3 | 8. ASB13 | 8. PIM2 |
| 9. MS4A1 | 9. CD8 | 9. CD22 | 9. TBET |
| 10. FOXP1 | 10. CD45RO | 10. LMO2 | 10. CD8 |

| **ABC DLBCL vs PMBL** | | | |
| --- | --- | --- | --- |
| **Principal Component 1** | | **Principal Component 2** | |
| *Positive* | *Negative* | *Positive* | *Negative* |
| 1. CYB5R2 | 1. BAFF | 1. LMO2 | 1. TCRβ |
| 2. FOXP1 | 2. CD3 | 2. NEK6 | 2. CCDC50 |
| 3. LIMD1 | 3. CCND1 | 3. CD95 | 3. Iμ-Cμ |
| 4. CXCR5 | 4. MAML3 | 4. S1PR2 | 4. CD28 |
| 5. PIM2 | 5. NEK6 | 5. IL4I1 | 5. BCL2#1 |
| 6. CD71 | 6. CD4 | 6. TRAF1 | 6. IGHM |
| 7. IRF4 | 7. CD28 | 7. CD40 | 7. ICOS |
| 8. RAB7L1 | 8. APRIL | 8. MS4A1 | 8. FOXP1 |
| 9. MYC#1 | 9. CD8 | 9. PDL1 | 9. CD3 |
| 10. BCL2#1 | 10. S1PR2 | 10. CD23 | 10. FOXP3 |

| **GCB DLBCL vs PMBL** | | | |
| --- | --- | --- | --- |
| **Principal Component 1** | | **Principal Component 2** | |
| *Positive* | *Negative* | *Positive* | *Negative* |
| 1. CD10 | 1. PRF | 1. IL4I1 | 1. TCRβ |
| 2. KI67 | 2. CD3 | 2. CD23 | 2. FOXP3 |
| 3. MS4A1 | 3. BAFF | 3. PDL2 | 3. CD28 |
| 4. MYBL1 | 4. CCND2 | 4. PDL1 | 4. CD3 |
| 5. BCL6#1 | 5. TBET | 5. NEK6 | 5. TCRα |
| 6. XPOWT | 6. GRB | 6. TRAF1 | 6. CD5 |
| 7. TCL1A | 7. CD8 | 7. CD95 | 7. ICOS |
| 8. CD22 | 8. CD19 | 8. MAL | 8. GATA3 |
| 9. CRBN | 9. CCND1 | 9. ALK | 9. CD27 |
| 10. FOXP1 | 10. LAG3 | 10. S1PR2 | 10. CTLA4 |

| **GCB DLBCL vs FL** | | | |
| --- | --- | --- | --- |
| **Principal Component 1** | | **Principal Component 2** | |
| *Positive* | *Negative* | *Positive* | *Negative* |
| 1. Ki67 | 1. CD3 | 1. PDL2 | 1. ICOS |
| 2. CD10 | 2. GATA3 | 2. BAFF | 2. MS4A1 |
| 3. XPOWT | 3. CD40L#1 | 3. CD68 | 3. BANK |
| 4. MYBL1 | 4. CD28 | 4. CD4 | 4. CD23 |
| 5. BCL6#1 | 5. CTLA4 | 5. PDL1 | 5. FOXP1 |
| 6. NEK6 | 6. CCND2 | 6. CCND1 | 6. CD28 |
| 7. BCMA | 7. CD5 | 7. APRIL | 7. CCDC50 |
| 8. BCL6#2 | 8. ICOS | 8. GRB | 8. CD40L#2 |
| 9. CD38 | 9. CCR4 | 9. PRF | 9. CD40L#1 |
| 10. CD22 | 10. FOXP3 | 10. FGFR1 | 10. Iε-Cε |

| **DLBCL vs Small cell lymphoma** | | | |
| --- | --- | --- | --- |
| **Principal Component 1** | | **Principal Component 2** | |
| *Positive* | *Negative* | *Positive* | *Negative* |
| 1. CYB5R2 | 1. CD3 | 1. S1PR2 | 1. CD5 |
| 2. LIMD1 | 2. CD28 | 2. CD68 | 2. TCRβ |
| 3. CXCR5 | 3. BAFF | 3. LMO2 | 3. GATA3 |
| 4. PIM2 | 4. ICOS | 4. BCL6#2 | 4. Iμ-Cμ |
| 5. IRF4 | 5. GATA3 | 5. Ki67 | 5. SH3BP5 |
| 6. MYD88e3-e4 | 6. CD45RO | 6. IL4I1 | 6. ZAP70 |
| 7. RAB7L1 | 7. CD4 | 7. NEK6 | 7. IGHD |
| 8. TACI | 8. CD8 | 8. CD86 | 8. CCND2 |
| 9. MS4A1 | 9. TCRγ | 9. BCL6#1 | 9. FOXP1 |
| 10. AID#1 | 10. CD40L#1 | 10. MAML3 | 10. IGHM |

| **FL vs Other small cell lymphoma** (SLL, MCL, MZL group) | | | |
| --- | --- | --- | --- |
| **Principal Component 1** | | **Principal Component 2** | |
| *Positive* | *Negative* | *Positive* | *Negative* |
| 1. LIMD1 | 1. ICOS | 1. MS4A1 | 1. GATA3 |
| 2. CCND2 | 2. CD28 | 2. CD40 | 2. PD1 |
| 3. STAT6 | 3 LMO2 | 3. B2M | 3. ZAP70 |
| 4. CCND1 | 4. CD10 | 4. BANK | 4. CD8 |
| 5. Iγ-Cγ | 5. BCL6#2 | 5. DUSP22 | 5. FGFR1 |
| 6. CD80 | 6. CTLA4 | 6. CD86 | 6. CD4 |
| 7. CREB3L2 | 7. CD45RO | 7. CCDC50 | 7. TBET |
| 8. CXCR5 | 8. MYBL1 | 8. KI67 | 8. CD3 |
| 9. IGHD | 9. AID#2 | 9. CD71 | 9. CD30 |
| 10. Iμ-Cμ | 10. BCL6#1 | 10. TCL1A | 10. STAT6 |

## Supplemental Table 3 Significantly overexpressed genes and corresponding E-values for each Volcano plot

| **ABC DLBCL vs GCB DLBCL** | | | |
| --- | --- | --- | --- |
| **Overexpressed in ABC** | **E-value** | **Overexpressed in GCB** | **E-value** |
| *IRF4* | 1.51E-21 | *NEK6* | 1.75E-15 |
| *LIMD1* | 1.11E-17 | *ASB13* | 2.27E-13 |
| *FOXP1* | 9.06E-17 | *MAML3* | 1.67E-12 |
| *PIM2* | 2.01E-14 | *S1PR2* | 3.66E-12 |
| *CREB3L2* | 2.63E-13 | *MYBL1* | 7.41E-10 |
| *TACI* | 1.68E-12 | *CD10* | 9.83E-09 |
| *RAB7L1* | 6.70E-12 | *SERPINA9* | 9.41E-08 |
| *CYB5R2* | 2.43E-10 | *BCL6#1* | 1.00E-07 |
| *CCND2* | 6.07E-08 | *ITPKB* | 7.49E-07 |
| *CCDC50* | 9.51E-08 | *LMO2* | 1.81E-06 |
| *SH3BP5* | 2.36E-07 | *BCL6#2* | 2.22E-05 |
| *IGHM* | 2.41E-07 | *CD38* | 7.33E-05 |
| *CCR7* | 5.89E-06 | *FOXP3* | 7.72E-05 |
| *PRDM1* | 2.25E-03 |  |  |
| *JH-Cμ* | 2.23E-02 |  |  |
| *AID#1* | 2.31E-02 |  |  |
| *AID#2* | 4.03E-02 |  |  |
| *CARD11* | 4.82E-02 |  |  |

| **ABC DLBCL vs PMBL** | | | |
| --- | --- | --- | --- |
| **Overexpressed in ABC** | **E-value** | **Overexpressed in PMBL** | **E-value** |
| *FOXP1* | 5.86E-21 | *BAFF* | 8.74E-08 |
| *PIM2* | 3.65E-15 | *CCND1* | 4.19E-07 |
| *TACI* | 2.30E-14 | *TRAF1* | 7.54E-07 |
| *IGHM* | 4.57E-14 | *NEK6* | 9.66E-07 |
| *IRF4* | 1.13E-13 | *LMO2* | 3.93E-06 |
| *BCL2#1* | 3.00E-13 | *CD95* | 4.14E-06 |
| *BCL2#2* | 4.79E-12 | *IL4I1* | 1.13E-04 |
| *LIMD1* | 5.51E-12 | *MAML3* | 2.04E-04 |
| *CREB3L2* | 3.86E-11 | *JAK2* | 3.41E-04 |
| *CXCR5* | 3.71E-10 | *CD86* | 5.76E-04 |
| *CYB5R2* | 6.62E-10 | *PDL2* | 6.82E-04 |
| *SH3BP5* | 9.13E-10 | *S1PR2* | 1.51E-03 |
| *TCL1A* | 2.33E-09 | *ITPKB* | 2.20E-03 |
| *BANK* | 4.10E-09 | *CD40L#1* | 5.02E-03 |
| *MYC#1* | 1.91E-08 | *ASB13* | 5.42E-03 |
| *CARD11* | 1.38E-07 | *MYBL1* | 6.43E-03 |
| *RAB7L1* | 2.64E-07 | *FGFR1* | 2.43E-02 |
| *JH-Cμ* | 4.48E-05 |  |  |
| *CCND2* | 5.41E-05 |  |  |
| *Iγ-Cγ* | 6.91E-05 |  |  |
| *CD71* | 1.15E-04 |  |  |
| *MYC#2* | 4.94E-02 |  |  |

| **GCB DLBCL vs PMBL** | | | |
| --- | --- | --- | --- |
| **Overexpressed in GCB** | **E-value** | **Overexpressed in PMBL** | **E-value** |
| *CARD11* | 3.54E-10 | *BAFF* | 3.12E-06 |
| *CXCR5* | 2.84E-09 | *PDL1* | 2.15E-05 |
| *BANK* | 1.98E-08 | *CD95* | 9.82E-05 |
| *CD27* | 2.29E-07 | *TRAF1* | 1.07E-04 |
| *BCL2#1* | 3.71E-07 | *JAK2* | 1.20E-04 |
| *TCL1A* | 3.99E-07 | *PDL2* | 6.70E-04 |
| *CD22* | 1.03E-06 | *IL4I1* | 1.26E-03 |
| *SERPINA9* | 6.11E-06 | *CCR7* | 2.07E-03 |
| *IGHM* | 3.00E-05 |  |  |
| *CD10* | 1.02E-04 |  |  |
| *BCL6#2* | 1.20E-03 |  |  |
| *TACI* | 3.81E-03 |  |  |
| *JH-Cμ* | 9.00E-03 |  |  |
| *IGHD* | 1.07E-02 |  |  |
| *MEF2B* | 1.37E-02 |  |  |
| *BCL6#1* | 1.67E-02 |  |  |

| **GCB DLBCL vs FL** | | | |
| --- | --- | --- | --- |
| **Overexpressed in GCB** | **E-value** | **Overexpressed in FL** | **E-value** |
| *CD68* | 9.65E-17 | *ICOS* | 2.41E-09 |
| *S1PR2* | 1.24E-12 | *CD40L#1* | 4.68E-09 |
| *KI67* | 1.39E-12 | *CD28* | 1.13E-08 |
| *IL4I1* | 3.64E-06 | *GATA3* | 4.10E-04 |
| *MAML3* | 4.56E-06 | *CXCL13* | 5.80E-03 |
| *PDL2* | 5.66E-06 |  |  |
| *CD163* | 1.47E-05 |  |  |
| *PDL1* | 3.38E-05 |  |  |
| *ASB13* | 1.54E-04 |  |  |
| *MYC#1* | 3.16E-04 |  |  |
| *CD70* | 4.05E-04 |  |  |
| *GRB* | 1.39E-03 |  |  |
| *AID#1* | 3.00E-03 |  |  |

| **DLBCL vs Small cell lymphoma** | | | |
| --- | --- | --- | --- |
| **Overexpressed in DLBCL** | **E-value** | **Overexpressed in Small Cell Lymphoma** | **E-value** |
| *CD68* | 1.08E-46 | *BANK* | 8.14E-15 |
| *BAFF* | 2.45E-24 | *CD40L#1* | 1.32E-12 |
| *CD163* | 1.96E-23 | *ICOS* | 4.59E-10 |
| *KI67* | 6.73E-20 | *CRBN* | 6.97E-10 |
| *S1PR2* | 8.07E-19 | *CD19* | 1.34E-09 |
| *IL4I1* | 1.51E-18 | *CD5* | 3.21E-09 |
| *RAB7L1* | 2.36E-14 | *CCDC50* | 9.17E-07 |
| *AID#2* | 1.08E-13 | *Iμ-Cμ* | 4.44E-06 |
| *AID#1* | 1.79E-13 | *CD23* | 1.95E-03 |
| *CYB5R2* | 1.51E-12 | *CCND2* | 2.80E-03 |
| *PRF* | 2.18E-12 | *IGHD* | 2.99E-03 |
| *CD71* | 2.50E-12 | *CCND1* | 6.11E-03 |
| *PIM2* | 9.41E-09 | *Iγ-Cγ* | 9.26E-03 |
| *GRB* | 2.05E-08 |  |  |
| *PDL2* | 5.96E-08 |  |  |
| *LMO2* | 3.73E-07 |  |  |
| *MAML3* | 3.78E-07 |  |  |
| *CD30* | 3.08E-05 |  |  |

| **FL vs Other small cell lymphomas** (SLL, MCL, MZL group) | | | |
| --- | --- | --- | --- |
| **Overexpressed in FL** | **E-value** | **Overexpressed in other small cell lymphoma** | **E-value** |
| *LMO2* | 6.87E-08 | *LIMD1* | 2.39E-16 |
| *BCL6#2* | 2.63E-07 | *CREB3L2* | 1.68E-12 |
| *CD10* | 1.19E-06 | *TACI* | 1.12E-09 |
| *BCL6#1* | 5.28E-06 | *IGHM* | 5.94E-09 |
| *CD28* | 1.11E-05 | *CD19* | 2.48E-08 |
| *ICOS* | 2.27E-05 | *SH3BP5* | 1.61E-07 |
| *MYBL1* | 3.36E-05 | *STAT6* | 2.87E-07 |
| *SERPINA9* | 5.62E-03 | *Iμ-Cμ* | 6.60E-07 |
|  |  | *CCDC50* | 7.68E-07 |
|  |  | *BANK* | 4.75E-06 |
|  |  | *IRF4* | 7.05E-06 |
|  |  | *CARD11* | 7.41E-06 |
|  |  | *IGHD* | 1.29E-05 |
|  |  | *Iγ-Cγ* | 3.17E-05 |
|  |  | *TBET* | 4.13E-05 |
|  |  | *CD5* | 5.86E-05 |
|  |  | *CCND2* | 2.27E-04 |
|  |  | *FGFR1* | 3.06E-04 |
|  |  | *CCND1* | 2.39E-03 |
|  |  | *FOXP1* | 3.03E-03 |
|  |  | *CD70* | 4.94E-03 |
|  |  | *JH-Cμ* | 7.63E-03 |
|  |  | *MYC#1* | 1.68E-02 |

## Supplemental Table 4 Clinical and Biological characteristics of local DLBCL cases according to MYCBCL2+ status

| **Characteristic** | **MYC/BCL2+ Double Expressor** | **non-Double Expressor** | **p-value** |
| --- | --- | --- | --- |
| All | 28 | 106 |  |
| **Age, years** |  |  |  |
| Median (range) | 73 (36-87) | 64 (19-87) |  |
| ≤ 60 years | 4 | 46 | **0.0043** |
| > 60 years | 24 | 60 |  |
| **Sex** |  |  |  |
| Female | 13 | 60 | 0.454 |
| Male | 15 | 46 |  |
| **Extralymphatic involvment >1** |  |  |  |
| No | 17 | 69 | 0.835 |
| Yes | 11 | 37 |  |
| **Stage** |  |  |  |
| I-II | 7 | 32 | 0.761 |
| III-IV | 21 | 74 |  |
| **B symptoms** |  |  |  |
| No | 18 | 66 | 1 |
| Yes | 10 | 40 |  |
| **Bulky disease (>10 cm)** |  |  |  |
| No | 18 | 66 | 1 |
| Yes | 10 | 40 |  |
| **Bone Marrow involvment** |  |  |  |
| No | 22 | 94 | 0.23 |
| Yes | 6 | 12 |  |
| **LDH** |  |  |  |
| Normal | 20 | 93 | **0.044** |
| High | 8 | 13 |  |
| **ECOG** |  |  |  |
| 0-1 | 19 | 87 | 0.279 |
| ≥ 2 | 8 | 19 |  |
| **IPI** |  |  |  |
| 0-2 | 8 | 53 | 0.07 |
| 3-5 | 20 | 53 |  |
| **Cell of Origin according to the assay** |  |  |  |
| ABC | 20 | 33 | **<0.0001** |
| GCB | 8 | 43 |  |
| PMBL | 0 | 30 |  |

# Supplemental Figures

## Supplemental Figure 1 Comparison of Nanostring nCounter and gene expression data

Gene expression data were compared with raw Nanostring nCounter data (Nanostring Technologies, Seattle, Washington) obtained from 96 samples. Gene expression data were normalized to allow comparisons between individual genes. Significant correlations were obtained for all 15 markers from the nCounter Lymph2Cx assay, showing a strong agreement between the two methods. Student's t test statistic and Spearman's rank correlation coefficient were used to analyze the data.


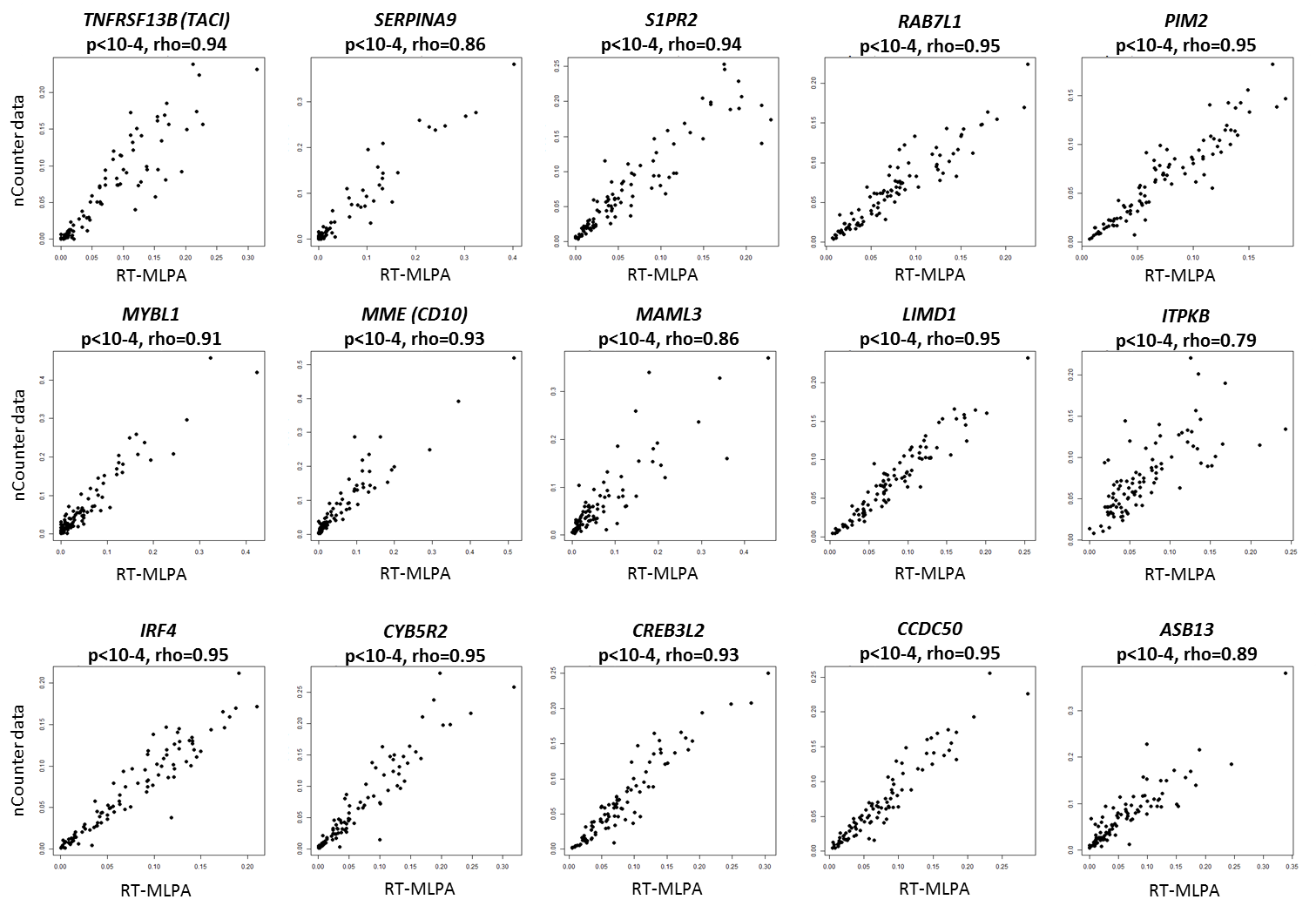


## Supplemental Figure 2 Comparison of IHC results and gene expression data

**A.** Gene expression data for the markers from the Hans algorithm (*CD10*, *BCL6* and *IRF4/MUM1*), the proliferation marker *Ki67* and the *BCL2* and *MYC* prognostic markers were compared with IHC staining in 48 DLBCL samples from a clinical trial with centralized review. Significantly higher expression in GEP was observed in samples considered positive using IHC, for all markers, showing that this assay can be used as an alternative to evaluate these markers.


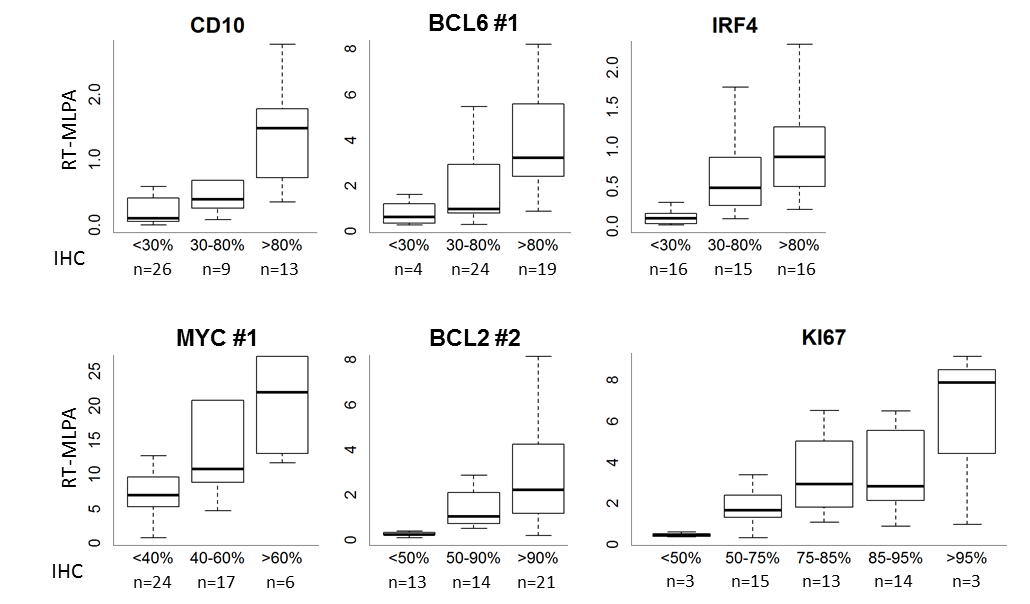


## Supplemental Figure 3 Transcriptomic expression of the markers from the ABC and GCB signatures in DLBCL

Differential expression of the markers from the ABC and GCB signature those are useful for distinguishing ABC from GCB DLBCL. **** p<10^-4^ by the Wilcoxon test.


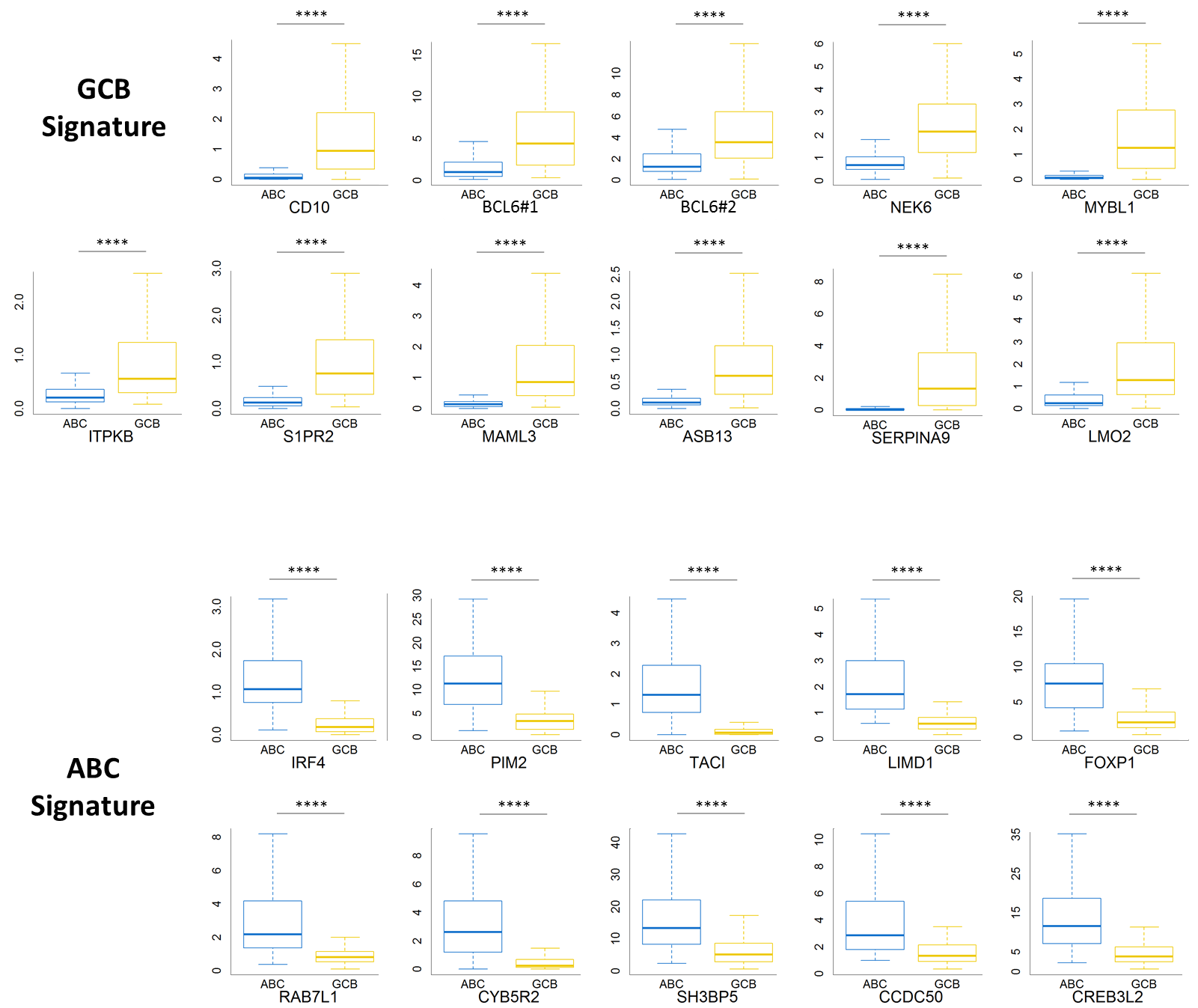


## Supplemental Figure 4 Schematic overview of the study design

For details on the clinical characteristics and pathological features of the patients, refer to Supplemental Tables 1 and 4.


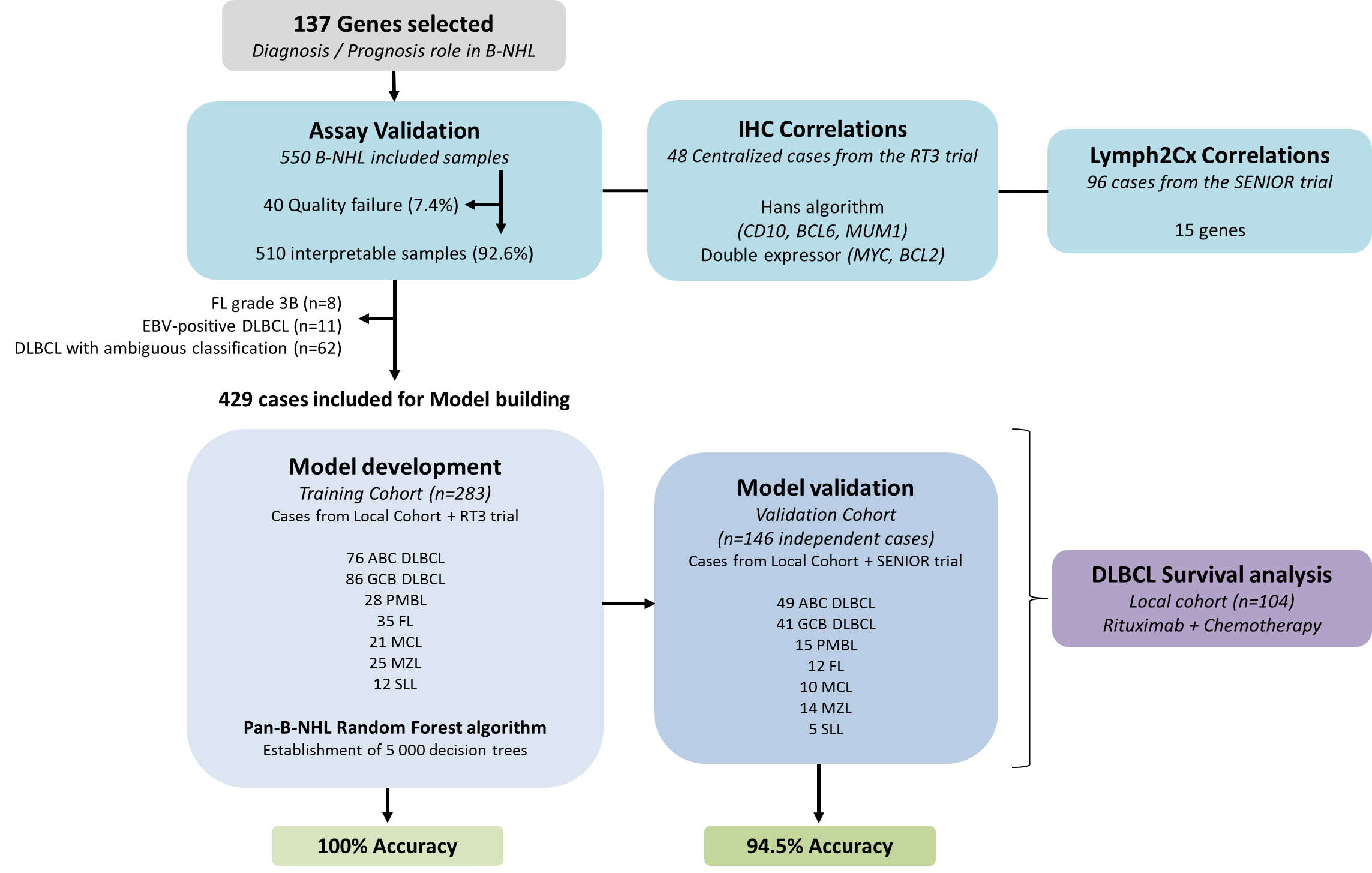


## Supplemental Figure 5 Progression-free survival (PFS) and overall survival (OS) in patients with DLBCL treated with rituximab plus chemotherapy from a local cohort stratified according to GCB/ABC/PMBL cell-of-origin

Survival curves for 104 patients from the local cohort stratified according to GCB, ABC or PMBL cell-of-origin determined by the random forest predictor


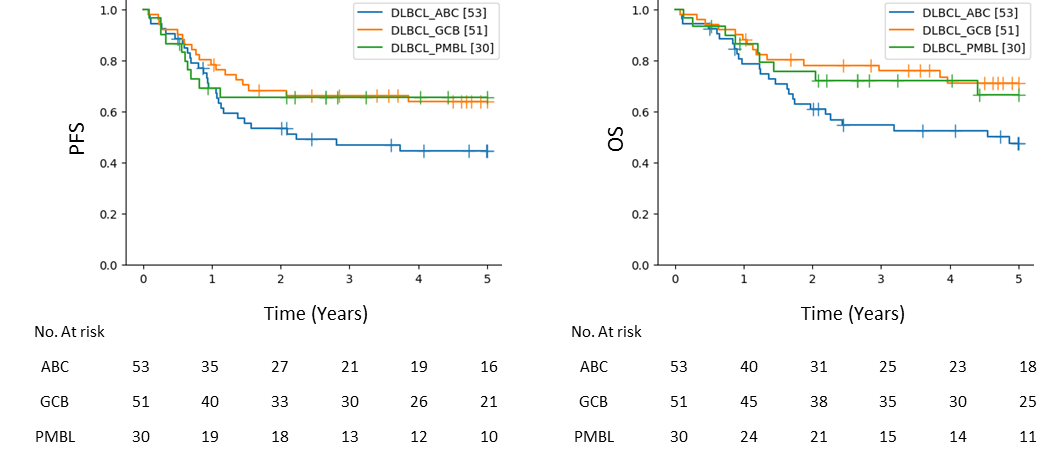


## Supplemental Figure 6 Progression-free survival (PFS) and overall survival (OS) of patients with DLBCL treated with rituximab plus chemotherapy from a local cohort stratified according to CARD11, CREB3L2, STAT6 and CD30 expression

Survival curves for 104 patients from the local cohort stratified according to the **A.** CARD11 status, **B.** CREB3L2 status, **C.** STAT6 status, or **D.** CD30 status.


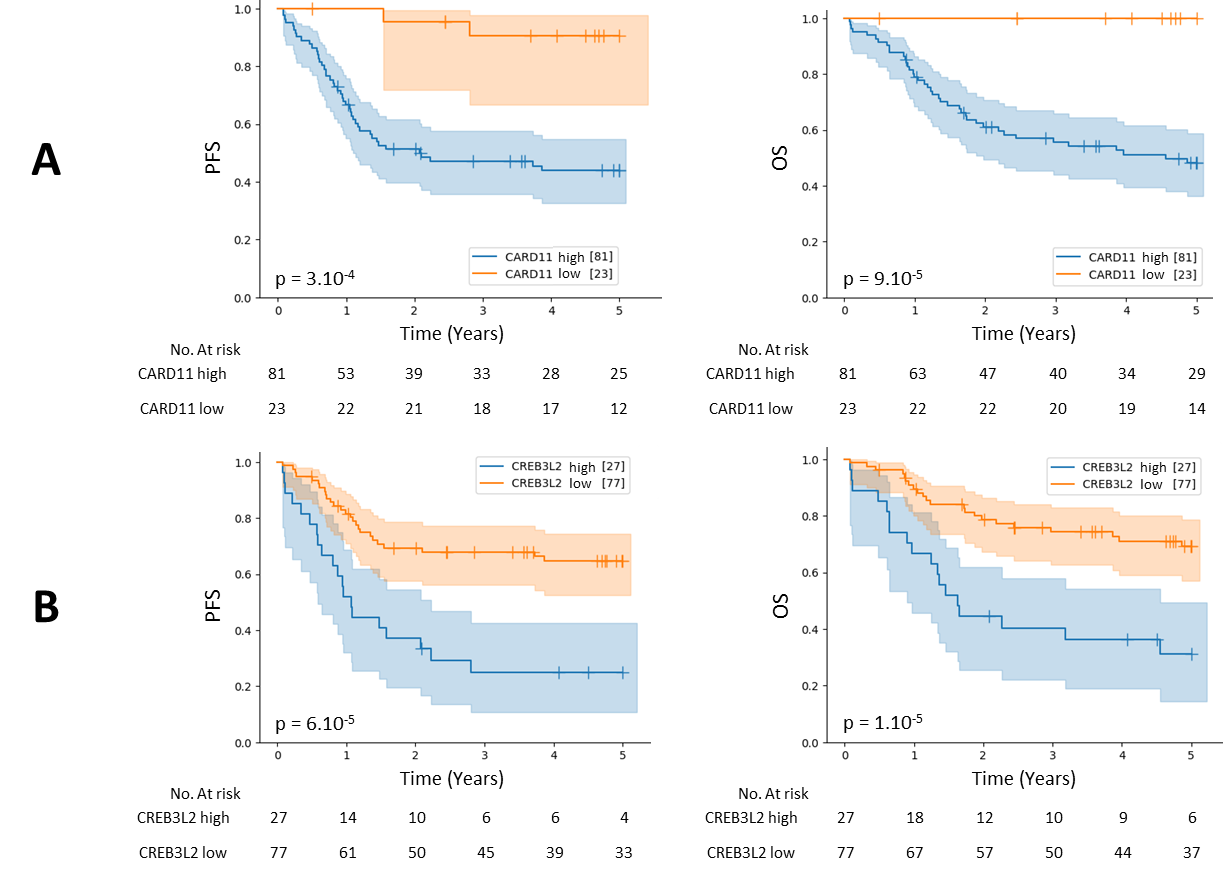

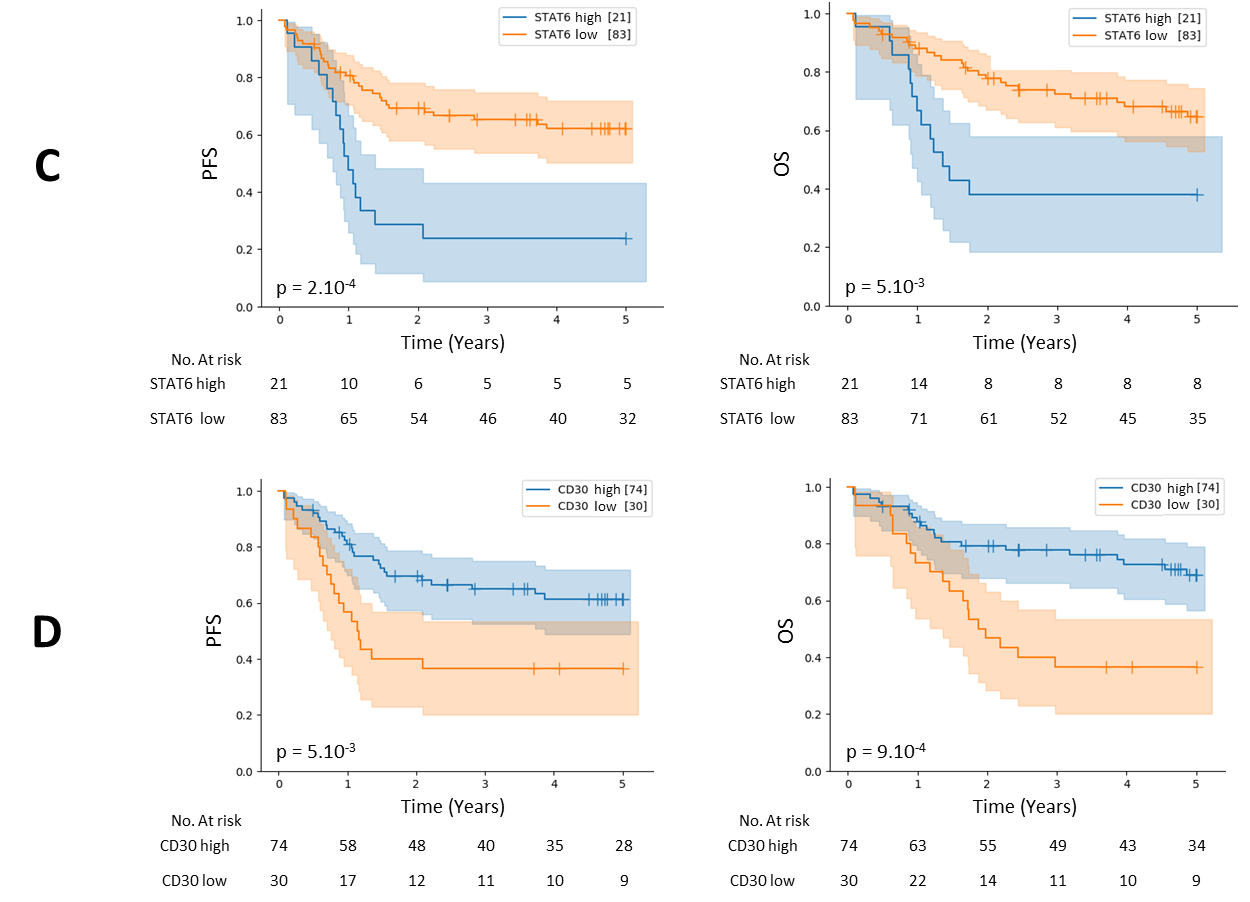

Supplement: Supplementary file 1 — Supplementary information [file 41408_2020_322_MOESM1_ESM.docx]
